# Hemophagocytic Lymphohistiocytosis and Infection: A Literature Review

**DOI:** 10.7759/cureus.22411

**Published:** 2022-02-20

**Authors:** Evgenia Koumadoraki, Nikolaos Madouros, Shayka Sharif, Amber Saleem, Sommer Jarvis, Safeera Khan

**Affiliations:** 1 Pathology, California Institute of Behavioral Neurosciences & Psychology, Fairfield, USA; 2 Surgery, California Institute of Behavioral Neurosciences & Psychology, Fairfield, USA; 3 Internal Medicine, California Institute of Behavioral Neurosciences & Psychology, Fairfield, USA; 4 Family Medicine, California Institute of Behavioral Neurosciences & Psychology, Fairfield, USA; 5 Anatomy/Cell Biology, California Institute of Behavioral Neurosciences & Psychology, Fairfield, USA

**Keywords:** hemophagocytic syndrome, infection, lymphohistiocytosis, hemophagocytic, ebv complications

## Abstract

Hemophagocytic lymphocytosis (HLH) is a life-threatening, underdiagnosed syndrome caused by the excessive release of inflammatory mediators. Primary lymphocytosis is usually seen in young children and is associated with genetic defects, while secondary lymphocytosis is presented in adults due to malignancy, rheumatic disease, or infection. The aim of this study is to describe the infectious agents that trigger HLH in the adult population and provide diagnostic and treatment guidelines for this life-threatening syndrome. We conducted a literature review using PubMed as our basic database. We collected papers from the past six years that studied infectious agents that triggered HLH and described the most recommended treatment options for this serious condition. A total of 32 studies were included for this literature review.HLH is considered a syndrome with variable symptoms, and clinicians should be familiar with its complexity and the pathologies that could contribute to its presentation. Collaboration between physicians and awareness are basic steps for the management of patients with HLH.

## Introduction and background

Hemophagocytic lymphohistiocytosis (HLH) is a severe life-threatening syndrome caused by an overwhelming inflammatory response and excessive immune cell activation. This inflammatory process is associated with uncontrolled macrophage activation and the release of a large number of cytokines [1], responsible for this heterogeneous syndrome's clinical manifestations, the main features of which include fever, hepatosplenomegaly, pancytopenia, and coagulopathy. Macrophages and T cells infiltrate target organs, such as the liver, the spleen, the brain, and specifically the bone marrow, to promote hemophagocytosis [1]. HLH is classified into primary HLH, which has a genetic background, and secondary HLH, which is an acquired condition mostly related to infections, malignancies, or autoimmune conditions.

**Figure 1:**
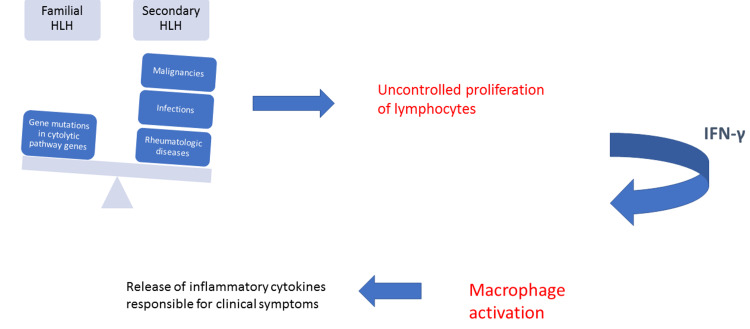
Hemophagocytic syndrome pathogenesis Original illustration HLH: hemophagocytic lymphohistiocytosis

Primary HLH, also known as familial HLH, is usually onset in childhood and is caused by genetic defects in lymphocyte cytolytic activity. Normally, CD8+ lymphocytes and natural killer (NK) cells are responsible for antigen-independent lysis of target cells (malignant, infected) by perforins that create holes in the cell membrane and granzymes that enter through these pores and promote apoptosis. Mutations in the genes that code for proteins involved in the cytolytic pathway [2] promote sustained inflammation and excessive cytokine production, such as IFN-γ, IL-6, and IL-18 [3], which contribute to the clinical presentation of the syndrome as shown in Figure 1. Other rare genetic disorders, such as Chediak Higashi (associated with *LYST* gene mutation) and Griscelli syndrome, have also been noted to trigger HLH [2].

On the other hand, secondary HLH is an acquired condition usually associated with viral, bacterial, fungal, and parasitic infections, such as leishmaniasis. Viral infections are the leading cause of secondary HLH, and Epstein-Barr virus (EBV) is the most common HLH-associated virus. In addition, secondary HLH is observed as a complication of malignancies, metabolic disturbances, and rheumatic diseases, such as juvenile arthritis or systemic lupus erythematosus. As far as rheumatic diseases are concerned, HLH is known as macrophage activation syndrome [4]. The HLH in adults is an underdiagnosed condition with a variety of symptoms; hence, the exact pathophysiological mechanism that leads to a sustained inflammatory response is not entirely understood [4]. In addition, even though the diagnostic criteria are specific and easily applied to all pediatric patients, in adults, there are multiple diagnostic challenges to be considered and other diseases with similar symptoms to be excluded.

Our study tries to raise the awareness of the clinicians for this underdiagnosed condition that leads to multiorgan failure and lethal complications and, therefore, it should be considered as a medical emergency that requires immediate treatment. The purpose of this review is to provide further information about the pathogenesis of HLH related to infectious agents, describe the symptoms and the criteria that should raise awareness of this heterogeneous syndrome, and build an evidence base for better treatment.

## Review

Methods

A thorough literature search was performed via PubMed for relevant published studies, using as keywords "Hemophagocytic lymphohistiocytosis and infection." Articles were also collected from Google Scholar and Cochrane library. We selected articles from the past six years written in the English language that referred to adult human beings exclusively. Also, we excluded articles that were case reports and papers without an available abstract. We applied the inclusion and exclusion criteria and removed duplicate publications. References were also checked for articles that may be relevant to our topic. The study was designed as a literature review so no statistical analysis was conducted.

Results

We included 23 articles from our PubMed search after scrupulous analysis that fulfilled our inclusion and exclusion criteria. We also added six papers that contained useful information related to our topic from references and two other studies from other sources. Since this review aims to provide further information about the infectious agents that trigger HLH in adults, we collected those studies that contained a specific number of patients with HLH. We categorized them according to their symptoms and outcome disorders that led to this inflammatory response. We focused on those patients whose HLH was triggered by infectious agents. Overall, we collected 18 studies with a total number of 636 patients with infection-triggered HLH. Interestingly, most cases were related to viral infections, and EBV, in particular, accounted for more than half of the cases. The remaining papers were used to collect information about the diagnosis and treatment of secondary HLH, two demanding fields studied in our review paper.

Discussion

Diagnostic Challenges in Secondary HLH

HLH is a complex condition caused by an excessive response of immune cells to a specific trigger and multiple cytokines release. Primary HLH is usually seen in infants and in children less than two years of age and is caused by specific gene mutations in the perforin-mediated cytolytic pathway, which is used by NK and CD8 cells. Secondary HLH, on the other hand, is presented in adults and is related to infections (mainly EBV) and malignancies, especially to T-cell lymphomas. HLH is also associated with autoimmune diseases and rare metabolic conditions, including lysinuric protein intolerance and Wolman's disease [2].

HLH is a clinical entity with signs of systematic illnesses and a variety of symptoms, the most common of which are:

Fever: it is one of the most common symptoms of HLH. The overproduction of IL1 causes it, and even though it seems a quite sensitive marker for HLH, it is not specific, and multiple other clinical conditions should be excluded.

Splenomegaly: It is caused by infiltration of lymphocytes and macrophages; however, like fever, it is present in many other conditions such as lymphoma, portal hypertension, myeloproliferative diseases, etc.

Cytopenias: According to studies, thrombocytopenia seems the most common symptom identified in 78% of adult cases [5]. Also, anemia and neutropenia are found in 67% and 42%, respectively [5]

Hemophagocytosis: It is a histopathological finding that can occasionally be identified in the bone marrow, the spleen, the liver, and the lymph nodes. It depicts the hemophagocytosis of hematopoietic cells by activated macrophages. Even though it is a distinctive feature of HLH, this finding is present in 25-100% of cases [6]. Hence, hemophagocytosis is not required for the diagnosis of HLH. In many patients, this histopathological finding may be absent in the early stages of the disease, and it is discovered in the further course [2].

Ferritin: Elevated ferritin levels of this iron-storing protein have been associated with HLH, especially when its concentration is higher than 500 μg/L. In a review of adult patients with HLH, ferritin concentration was >500 in 90% of patients, >1000 in 71% of patients, and >10.000 μg/L in just 24% of patients [5] . Also, in a single retrospective study of serum ferritin, levels >10.000 μg/L were 96% specific and 90% sensitive for HLH [7] . However, ferritin levels could be elevated in multiple other conditions, such as chronic renal failure and malignancies, making it demanding to identify the exact cause of elevated ferritin, especially in adults with chronic diseases.

Elevated IL2 receptor (soluble CD25): An elevated IL2 is a marker of activated lymphocytes that promote the immune response, and a CD25 level above 2400 U/ml is one of the criteria for the diagnosis of HLH [8] .

Other findings that are also associated with HLH are hypertriglyceridemia, hypofibrinogenemia, lymphadenopathy, and central nervous system (CNS) dysfunction.

Since the clinical presentation of HLH is very variable, and it may be similar to that of sepsis, septic shock, and other inflammatory procedures, the clinical suspicion should be high. The first criteria used for the diagnosis were proposed by the Familial Hemophagocytic Lymphohistiocytosis study group in 1991 and were based on pediatric patients under 15 years old. There are eight diagnostic criteria based on the ones proposed in 1991 and were revised in 2004 [9], of which five must be present for the diagnosis: (1) fever, (2) splenomegaly, (3) cytopenia in at least two lines with hemoglobin <90 g/L, neutrophil count less than 100*109 /L and platelet count less than 1*10 9/L, (4) hyperferritinemia >500μg/L 5) hypofibrinogenemia<1,5 g/L or hypertriglyceridemia >3mmol/L, (6) high soluble CD25 >2400U/ml, (7) hemophagocytosis in bone marrow, spleen, or lymph nodes, and (8) low or absent NK cell activity [9]. However, these criteria are based mostly on pediatric patients and their application in adults has limitations and further studies need to be made specifically for adult population

HLH and Infections

This literature review collected 18 recent studies with adult patients with HLH caused by infectious diseases. In these cases, the infectious triggers for HLH were mainly viruses (EBV and cytomegalovirus (CMV)), bacterial infections, such as *S.aureus*, *Mycobacterium tuberculosis*, or even parasites like Leishmania. Interestingly, most adult patients with HLH due to infections presented with fever, elevated ferritin levels, cytopenias in at least two cell lines, and elevated LDH [10-12]. Overall, patients with existing malignancy, lymphopenia, low albumin levels, and platelet count less than 20*109 had the worst prognosis [13,14].

EBV-associated HLH is the most common type of infection-associated lymphohistiocytosis. Interestingly, almost 50% of the 636 cases of infection-associated HLH from these studies were related to EBV infection. According to a large recent study with 133 EBV-related cases, HLH is a life-threatening condition with one-year mortality of 78% [15]. The study results showed that patients who had at least partial response and symptom improvement at the beginning of the treatment had a better prognosis than the patients with no remission after the initial treatment [15]. Another study by Yoon et al. suggested that patients with HLH caused by EBV responded faster to the initial treatment but presented high relapse rates; according to this study, 126 patients with infection triggered HLH unstable or no response in the first eight weeks of treatment, age >45 years old, and low platelet count was associated with a worse prognosis [16]. Finally, in a retrospective analysis of 96 patients with HLH and CNS involvement, the presence of EBV infection itself proved to be a risk factor of CNS symptoms, regardless of which EBV19 infected cells [17].

Two other studies reviewed 71 patients with HLH related to *Mycobacterium tuberculosis* infection, suggesting that patients often presented with fever and hepatosplenomegaly [18,19]. In patients with tuberculosis (TB), hemophagocytic syndrome should be suspected in cases with cytopenias, coagulation disorders, and splenomegaly [18]. In fact, in some patients, these symptoms' manifestations were present before the diagnosis of TB [18]. Hence, it is crucial to immediately initiate anti-TB treatment after the definite diagnosis of TB for HLH in these patients to be prevented [19].

Another study that included 13 fatal cases of H1N1 influenza with HLH suggested that gene mutations predispose to HLH caused by H1N1 [20]. However, more extensive studies are required to determine whether there is a genetic background for those who suffer from infection caused by HLH. Three other studies tried to evaluate whether the cytokine storm observed in patients with coronavirus disease 2019 (COVID-19) is sufficient to cause HLH [21-23]. Interestingly, fever, hyperferritinemia, and elevated triglycerides were commonly described in patients with COVID-19 [21]. However, even though many patients with COVID=19 present these findings, only a few of them fulfill Histological Score (HScore) for HLH [22,23], suggesting that the current criteria might need modification and cannot be broadly implied.

Four other studies tried to evaluate the association between HIV and HLH [24-27]. A large study with 3066 patients with HIV noticed that the most common symptoms in immunocompromised patients with HLH were fever, cytopenias, and elevated ferritin. In contrast, 33% of the patients presented CNS involvement [24]. In addition, another study by Lerolle et al. suggested that in immunocompromised patients, HIV was considered a predisposing condition for hemophagocytic syndrome and was not the actual trigger for HLH [25]. However, HLH could be a presentation of acute HIV infection and it should be considered in cases when the cause of HLH is not apparent [26]. Interestingly, almost one-fourth of immunocompromised patients had concomitant infections like that of human herpesvirus-8 (HHV8), *Candida*, or *Pneumocystis jirovecii* [25]. Another retrospective study with 36 HIV patients showed that infections were the most common cause for HLH in those patients, and especially infectious agents like *Mycobacterium*, *Cryptococcus*, or CMV [27].

Treatment of Secondary HLH

Since HLH is caused by excessive release of inflammatory mediators, the mainstay of treatment is to decrease circulating cytokines and support organ failure. Broadly, the current treatment of HLH is based on the HLH-1994 and HLH-2004 protocols that include immunosuppressive regimens [28], such as dexamethasone, cyclosporine, and etoposide. Cyclosporine A depletes the actions of lymphocytes and interferes with the action of macrophages. Etoposide depletes activated T-cells. It should be used as a first-line treatment, especially in patients with severe organ damage [28]. The standard dose is approximately 150 to 200mg/m (dose reduction is necessary for patients with renal failure) [29]. However, etoposide is associated with secondary infections due to white blood cell depletion and secondary cancer, and a weekly evaluation of the need for continued etoposide therapy should be considered [28]. In addition, the administration of prophylactic treatment for fungal infections and *Pneumocystis jirovecii* should be given to high-risk patients with depleted leukocyte function due to HLH-treatment protocol [30].

Moreover, in infection-triggered HLH, a high dose of intravenous immunoglobulins should be considered in combination with steroids [2] Antimicrobial agents should also be immediately started at the presentation time. Interestingly, in some cases of HLH, due to specific agents, only the use of antimicrobial treatment is sufficient to treat HLH. For example, leishmaniasis HLH is cured with amphotericin B [30], while tuberculosis requires quadruple antibiotic treatment. Caution should be raised in patients with EBV-related HLH since many patients present with serious symptoms and high remission rates. A study that included 93 patients with EBV-HLH [31] concluded that in adult patients, the introduction of etoposide in the first four weeks of treatment resulted in a better prognosis, and early treatment with etoposide should be recommended. Apart from that, Rituximab's introduction is a CD-20 antibody that depletes B cell function, proved to be beneficial as adjuvant treatment [32] . However, it could not be used as monotherapy since it is affected in EBV-HLH, T, and NK cells. Another multicentre, non-randomized trial proved the efficacy of ruxolitinib combined with the doxorubicin‐etoposide‐methylprednisolone (Ru‐DEP) regimen as a salvage therapy for refractory/relapsed HLH [33]. Interestingly, the majority of the patients who received this combination attained partial response and improvement of their laboratory tests.

Finally, supportive treatment should be offered to all patients, and severely ill ones should be transferred to the intensive care unit to be supported with mechanical ventilation and vasoactive drugs [34]. Coagulation disorders [34] should also be considered since many of these patients require red blood cells due to anemia, fresh frozen plasma due to coagulation disorders, and platelets due to thrombopenia.

Limitations

In this study we tried to provide information about several infectious agents that trigger HLH and summarized the diagnostic criteria and the basic treatment guidelines for HLH. However, as already mentioned, hemophagocytic syndrome in adults is not a well- studied medical entity and the diagnostic criteria that we provided may not be applicable to all patients. In addition, some included studies that described the connection of HLH with specific infectious agents, included a small number of patients. Moreover, we reviewed studies from the last six years, so some relevant studies from the previous years have been excluded by our criteria limitations.

## Conclusions

HLH is a hyperinflammatory condition caused by excessive release of cytokines . A familial form of HLH is presented in newborns and young children due to genetic predisposition. In contrast, secondary HLH in adults is related to many pathological processes and diseases, such as malignancy and infections. The clinical manifestations of the syndrome are variable, and clinicians should be aware of its symptoms and exclude other similar diseases. The current diagnostic is mainly from studies that include children and young adolescents; hence, there is a need for future studies that analyze the clinical manifestations specifically in adults. Following any diagnosis of HLH, immediate treatment is crucial and patients should be screened regularly for recurrences. The therapeutic strategy should be planned by a multidisciplinary team in order a high-quality,patient-centred approach to be achieved.

Even though the exact mechanism that triggers HLH is unclear , this study summarizes the basic principles available and mentions the clinical criteria for the diagnosis of this complex syndrome. By shreding some light in the possible infectious causes, this study would raise the awareness of the physicians for early recognition and initiation of treatment.
